# PHYSICAL FUNCTION, SELF-PERCEPTIONS OF AGING, AND DEPRESSIVE SYMPTOMS AMONG OLDER ADULTS

**DOI:** 10.1093/geroni/igac059.2009

**Published:** 2022-12-20

**Authors:** Suyoung Nah, Lynn Martire, Ashley Tate

**Affiliations:** Pennsylvania State University, State College, Pennsylvania, United States; Pennsylvania State University, University Park, Pennsylvania, United States; Pennsylvania State University, University Park, Pennsylvania, United States

## Abstract

Poor physical function has been linked to greater depressive symptoms among older adults. On the other hand, older adults’ perceptions of positive and negative age-related changes provide personal strength and vulnerability to stressful events, respectively. We therefore expected that positive self-perceptions of aging (SPA) would be associated with fewer depressive symptoms, while negative SPA would be related to greater depressive symptoms, beyond the effect of physical function. We further tested the hypotheses that positive SPA would buffer the association between physical function and greater depressive symptoms, whereas negative SPA would exacerbate this association. This study used data from 108 older adults (mean age = 81.09) in independent-living or retirement communities. Results from a linear regression revealed that more positive SPA (B = -0.21, p = .02) and less negative SPA (B = 0.21, p = .06) were associated with fewer depressive symptoms, even after controlling for physical function, both types of SPA, and other covariates. In contrast, physical function was no longer significantly associated with depressive symptoms (B = -0.15, p = .19), after controlling for both types of SPA. There were no significant moderating effects of positive and negative SPA. Findings suggest that how positively and negatively older adults perceive their own aging may be important for their mental health while experiencing less physical function in late life.

